# 2-[4-(Carb­oxy­meth­yl)phen­oxy]acetic acid

**DOI:** 10.1107/S1600536810051810

**Published:** 2010-12-18

**Authors:** Jun-Dan Fu, Yi-Hang Wen

**Affiliations:** a Zhejiang Key Laboratory for Reactive Chemistry on Solid Surfaces, Institute of Physical Chemistry, Zhejiang Normal University, Jinhua, Zhejiang 321004, People’s Republic of China

## Abstract

The title compound, C_10_H_10_O_5_, was obtained by the reaction of 4-hy­droxy­phenyl­acetic acid with chloro­acetic acid. In the crystal, the mol­ecules form a three-dimensional network by way of inter­molecular O—H⋯O hydrogen bonding.

## Related literature

For applications of metal-organic frameworks with carb­oxy­lic acid ligands, see: Kuppler *et al.* (2009) ▶; Jahan *et al.* (2010 ▶); Armelao *et al.* (2010 ▶); Yashima *et al.* (2009 ▶). The title compound was obtained by the reaction of 4-hy­droxy­phenyl­acetic (Gracin *et al.*, 2005 ▶) and chloro­acetic acid (Sandhu *et al.*, 1991 ▶).
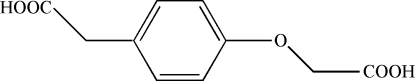


## Experimental

### 

#### Crystal data

C_10_H_10_O_5_
*M* *_r_* = 210.18
Orthorhombic, 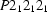

*a* = 5.4640 (2) Å
*b* = 10.7798 (5) Å
*c* = 16.2550 (7) Å
*V* = 957.43 (7) Å^3^
*Z* = 4
Mo *K*α radiation
μ = 0.12 mm^−1^
*T* = 296 K
0.42 × 0.38 × 0.31 mm

#### Data collection

Bruker APEXII area-detector diffractometer
Absorption correction: multi-scan (*SADABS*; Sheldrick, 1996 ▶) *T*_min_ = 0.95, *T*_max_ = 0.96
11814 measured reflections
1315 independent reflections
1162 reflections with *I* > 2σ(*I*)
*R*_int_ = 0.049

#### Refinement

*R*[*F* ^2^ > 2σ(*F* ^2^)] = 0.037
*wR*(*F* ^2^) = 0.099
*S* = 1.07
1315 reflections
136 parameters
H-atom parameters constrained
Δρ_max_ = 0.14 e Å^−3^
Δρ_min_ = −0.19 e Å^−3^

### 

Data collection: *APEX2* (Bruker, 2006 ▶); cell refinement: *SAINT* (Bruker, 2006 ▶); data reduction: *SAINT*; program(s) used to solve structure: *SHELXS97* (Sheldrick, 2008 ▶); program(s) used to refine structure: *SHELXL97* (Sheldrick, 2008 ▶); molecular graphics: *SHELXTL* (Sheldrick, 2008 ▶); software used to prepare material for publication: *SHELXTL*.

## Supplementary Material

Crystal structure: contains datablocks I, global. DOI: 10.1107/S1600536810051810/ds2074sup1.cif

Structure factors: contains datablocks I. DOI: 10.1107/S1600536810051810/ds2074Isup2.hkl

Additional supplementary materials:  crystallographic information; 3D view; checkCIF report

## Figures and Tables

**Table 1:** Hydrogen-bond geometry (Å, °)

*D*—H⋯*A*	*D*—H	H⋯*A*	*D*⋯*A*	*D*—H⋯*A*
O2—H2⋯O4^i^	0.82	1.81	2.617 (2)	168
O5—H5⋯O1^ii^	0.82	1.85	2.666 (2)	171

Symmetry codes: (i) 
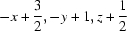
; (ii) 
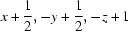
.

**Fig. 1.**
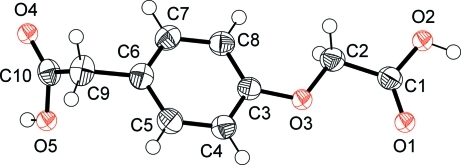
View of the molecular structure of (I), showing the atom-numbering scheme. Displacement ellipsoids are drawn at the 30% probability level.

**Fig. 2.**
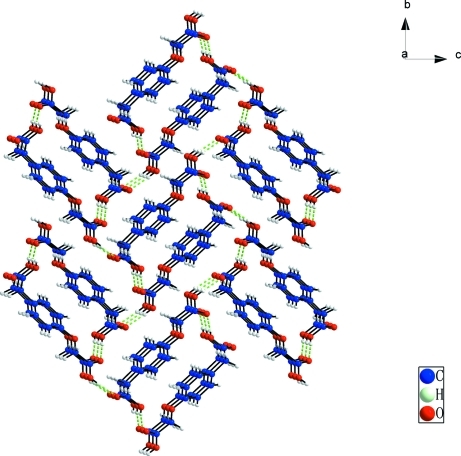
A packing diagram of (I), the hydrogen bonds are depicted as green dashed lines.

## supplementary crystallographic information

### Comment

Various aromatic carboxylic acid ligands are being used in designing  versatile metal-organic frameworks (MOFs) having potential applications (Kuppler *et al.*, 2009; Jahan *et al.*, 2010; Armelao *et al.*, 2010; Yashima *et al.*, 2009). In present work, we report the synthesis and single crystal structure of a new aromatic dicarboxylic acid ligand namely 4-carboxymethoxy phenylacetic acid (I) containing two flexible carboxylic acid groups, obtained by the reaction of 4-hydroxyphenylacetic (Gracin *et al.*, 2005) and chloroacetic acid (Sandhu *et al.*, 1991).

The molecular structure of the title compound is presented in Fig.1. The carboxymethoxy group is almost parallel to the benzene ring with a dihedral angle of 11.4 (1)° between the two least-squares planes,while the acetic acid unit is almost perpendicular to the benzene ring, making a dihedral angle of 72.4 (2)°.In addtion, the moleculars were linked by the intermolecular O—H···O hydrogen bonding forming a three-dimensional network (Fig.2).

### Experimental

The solution of 4-hydroxyphenylacetic acid (1.522 g, 10.0 mmol) in water (10 ml) neutralized with NaOH (0.8 g, 20 mmol) was added to a 1:1 mixture of chloroacetic acid (3.78 g, 40 mmol) and NaOH (1.600 g, 40 mmol) with stirring to adjust the pH value of the mixture to *ca* 11 and refluxed at 90 ^0^C for 3 h. The pH value was adjusted to 2–3 with concentrated hydrochloric acid. While cooling the mixture to room temperature, a white precipitate was appeared rapidly. The solid was filtrated, washed by water. Single crystals suitable for X-ray diffraction were obtained in the mother liquid after evaporation within a few days.

### Refinement

The Flack parameter could not be refined to an acceptable value as the title compound is a poor anomalous scatterer even after using MOVE command in Shelxl97. Therefore, the Friedel oppostites were merged (using MERG 3 command in Shelxl97) and Flack parameter is not reported. H-atoms were positioned geometrically and included in the refinement using a riding-model approximation [aromatic C–H = 0.93 Å, aliphatic C–H = 0.97 Å, O–H = 0.82 Å] with *U*_iso_(H) = 1.2*U*_eq_(C,*O*).

### Crystal data

**Table:**

C_10_H_10_O_5_	*F*(000) = 440
*M**_r_* = 210.18	*D*_x_ = 1.458 Mg m^−^^3^
Orthorhombic, *P*2_1_2_1_2_1_	Mo *K*α radiation, λ = 0.71073 Å
Hall symbol: P 2ac 2ab	Cell parameters from 6173 reflections
*a* = 5.4640 (2) Å	θ = 2.3–27.7°
*b* = 10.7798 (5) Å	µ = 0.12 mm^−^^1^
*c* = 16.2550 (7) Å	*T* = 296 K
*V* = 957.43 (7) Å^3^	Block, colourless
*Z* = 4	0.42 × 0.38 × 0.31 mm

### Data collection

**Table:**

Bruker APEXII area-detector diffractometer	1315 independent reflections
Radiation source: fine-focus sealed tube	1162 reflections with *I* > 2σ(*I*)
graphite	*R*_int_ = 0.049
phi and ω scans	θ_max_ = 27.7°, θ_min_ = 2.3°
Absorption correction: multi-scan (*SADABS*; Sheldrick, 1996)	*h* = −7→7
*T*_min_ = 0.95, *T*_max_ = 0.96	*k* = −13→14
11814 measured reflections	*l* = −20→21

### Refinement

**Table:**

Refinement on *F*^2^	Primary atom site location: structure-invariant direct methods
Least-squares matrix: full	Secondary atom site location: difference Fourier map
*R*[*F*^2^ > 2σ(*F*^2^)] = 0.037	Hydrogen site location: inferred from neighbouring sites
*wR*(*F*^2^) = 0.099	H-atom parameters constrained
*S* = 1.07	*w* = 1/[σ^2^(*F*_o_^2^) + (0.0425*P*)^2^ + 0.2342*P*] where *P* = (*F*_o_^2^ + 2*F*_c_^2^)/3
1315 reflections	(Δ/σ)_max_ < 0.001
136 parameters	Δρ_max_ = 0.14 e Å^−^^3^
0 restraints	Δρ_min_ = −0.19 e Å^−^^3^

### Special details

**Table:**

Geometry. All e.s.d.'s (except the e.s.d. in the dihedral angle between two l.s. planes) are estimated using the full covariance matrix. The cell e.s.d.'s are taken into account individually in the estimation of e.s.d.'s in distances, angles and torsion angles; correlations between e.s.d.'s in cell parameters are only used when they are defined by crystal symmetry. An approximate (isotropic) treatment of cell e.s.d.'s is used for estimating e.s.d.'s involving l.s. planes.
Refinement. Refinement of *F*^2^ against ALL reflections. The weighted *R*-factor *wR* and goodness of fit *S* are based on *F*^2^, conventional *R*-factors *R* are based on *F*, with *F* set to zero for negative *F*^2^. The threshold expression of *F*^2^ > σ(*F*^2^) is used only for calculating *R*-factors(gt) *etc*. and is not relevant to the choice of reflections for refinement. *R*-factors based on *F*^2^ are statistically about twice as large as those based on *F*, and *R*- factors based on ALL data will be even larger.

### Fractional atomic coordinates and isotropic or equivalent isotropic displacement parameters (Å^2^)

**Table:**

	*x*	*y*	*z*	*U*_iso_*/*U*_eq_	
O1	0.4626 (4)	0.67278 (16)	0.64905 (10)	0.0651 (5)	
O2	0.7484 (4)	0.79234 (17)	0.59564 (10)	0.0645 (5)	
H2	0.7132	0.8367	0.6349	0.077*	
O3	0.4929 (3)	0.51339 (14)	0.52607 (9)	0.0489 (4)	
O4	0.8002 (3)	0.06923 (15)	0.22690 (11)	0.0536 (4)	
O5	0.6100 (4)	−0.00299 (16)	0.33749 (9)	0.0589 (5)	
H5	0.7257	−0.0515	0.3374	0.071*	
C1	0.6127 (4)	0.6927 (2)	0.59700 (12)	0.0440 (5)	
C2	0.6695 (4)	0.6081 (2)	0.52593 (12)	0.0443 (5)	
H2A	0.6632	0.6538	0.4746	0.053*	
H2B	0.8320	0.5731	0.5320	0.053*	
C3	0.4856 (4)	0.43389 (19)	0.46020 (12)	0.0401 (4)	
C4	0.2906 (4)	0.3526 (2)	0.45967 (13)	0.0449 (5)	
H4A	0.1741	0.3559	0.5013	0.054*	
C5	0.2681 (4)	0.2661 (2)	0.39733 (13)	0.0467 (5)	
H5A	0.1357	0.2119	0.3972	0.056*	
C6	0.4405 (4)	0.2592 (2)	0.33498 (12)	0.0427 (5)	
C7	0.6354 (4)	0.3416 (2)	0.33640 (13)	0.0457 (5)	
H7A	0.7520	0.3381	0.2948	0.055*	
C8	0.6606 (4)	0.4294 (2)	0.39878 (12)	0.0445 (5)	
H8A	0.7925	0.4839	0.3991	0.053*	
C9	0.4187 (4)	0.1613 (2)	0.26811 (13)	0.0502 (5)	
H9A	0.2651	0.1171	0.2741	0.060*	
H9B	0.4198	0.2009	0.2145	0.060*	
C10	0.6279 (4)	0.07182 (19)	0.27412 (13)	0.0431 (5)	

### Atomic displacement parameters (Å^2^)

**Table:**

	*U*^11^	*U*^22^	*U*^33^	*U*^12^	*U*^13^	*U*^23^
O1	0.0757 (12)	0.0593 (11)	0.0602 (9)	−0.0208 (10)	0.0218 (10)	−0.0149 (8)
O2	0.0736 (12)	0.0572 (10)	0.0627 (10)	−0.0251 (10)	0.0144 (10)	−0.0129 (8)
O3	0.0598 (9)	0.0426 (8)	0.0444 (7)	−0.0102 (8)	0.0096 (8)	−0.0050 (6)
O4	0.0479 (8)	0.0490 (9)	0.0639 (9)	0.0009 (8)	0.0033 (8)	0.0069 (8)
O5	0.0625 (10)	0.0639 (10)	0.0503 (8)	0.0177 (10)	0.0026 (8)	0.0099 (8)
C1	0.0475 (11)	0.0409 (10)	0.0437 (10)	−0.0063 (10)	−0.0009 (10)	0.0025 (9)
C2	0.0446 (11)	0.0461 (11)	0.0423 (10)	−0.0056 (10)	0.0009 (10)	0.0003 (9)
C3	0.0448 (10)	0.0376 (9)	0.0378 (9)	0.0010 (9)	0.0000 (9)	0.0026 (8)
C4	0.0447 (11)	0.0452 (11)	0.0448 (10)	−0.0008 (10)	0.0034 (10)	0.0012 (9)
C5	0.0435 (11)	0.0452 (11)	0.0515 (11)	−0.0016 (10)	−0.0029 (10)	0.0017 (9)
C6	0.0436 (11)	0.0428 (10)	0.0416 (9)	0.0092 (10)	−0.0067 (9)	0.0012 (8)
C7	0.0436 (11)	0.0512 (12)	0.0422 (10)	0.0051 (10)	0.0030 (9)	0.0011 (9)
C8	0.0412 (10)	0.0465 (11)	0.0457 (10)	−0.0023 (10)	0.0002 (9)	0.0018 (9)
C9	0.0493 (12)	0.0541 (12)	0.0473 (11)	0.0129 (11)	−0.0122 (10)	−0.0060 (10)
C10	0.0455 (11)	0.0389 (10)	0.0451 (10)	0.0010 (10)	−0.0084 (10)	−0.0040 (9)

### Geometric parameters (Å, °)

**Table:**

O1—C1	1.198 (3)	C4—C5	1.383 (3)
O2—C1	1.305 (3)	C4—H4A	0.9300
O2—H2	0.8200	C5—C6	1.386 (3)
O3—C3	1.372 (2)	C5—H5A	0.9300
O3—C2	1.405 (3)	C6—C7	1.387 (3)
O4—C10	1.215 (3)	C6—C9	1.519 (3)
O5—C10	1.312 (3)	C7—C8	1.394 (3)
O5—H5	0.8200	C7—H7A	0.9300
C1—C2	1.504 (3)	C8—H8A	0.9300
C2—H2A	0.9700	C9—C10	1.499 (3)
C2—H2B	0.9700	C9—H9A	0.9700
C3—C4	1.380 (3)	C9—H9B	0.9700
C3—C8	1.383 (3)		
			
C1—O2—H2	109.5	C6—C5—H5A	119.6
C3—O3—C2	118.19 (16)	C5—C6—C7	118.39 (19)
C10—O5—H5	109.5	C5—C6—C9	120.5 (2)
O1—C1—O2	123.3 (2)	C7—C6—C9	121.11 (19)
O1—C1—C2	125.1 (2)	C6—C7—C8	121.5 (2)
O2—C1—C2	111.64 (18)	C6—C7—H7A	119.2
O3—C2—C1	107.31 (17)	C8—C7—H7A	119.2
O3—C2—H2A	110.3	C3—C8—C7	118.7 (2)
C1—C2—H2A	110.3	C3—C8—H8A	120.6
O3—C2—H2B	110.3	C7—C8—H8A	120.6
C1—C2—H2B	110.3	C10—C9—C6	109.89 (17)
H2A—C2—H2B	108.5	C10—C9—H9A	109.7
O3—C3—C4	115.09 (18)	C6—C9—H9A	109.7
O3—C3—C8	124.41 (19)	C10—C9—H9B	109.7
C4—C3—C8	120.47 (19)	C6—C9—H9B	109.7
C3—C4—C5	120.1 (2)	H9A—C9—H9B	108.2
C3—C4—H4A	119.9	O4—C10—O5	122.6 (2)
C5—C4—H4A	119.9	O4—C10—C9	124.4 (2)
C4—C5—C6	120.8 (2)	O5—C10—C9	112.97 (19)
C4—C5—H5A	119.6		
			
C3—O3—C2—C1	171.19 (18)	C5—C6—C7—C8	0.3 (3)
O1—C1—C2—O3	8.2 (3)	C9—C6—C7—C8	−178.2 (2)
O2—C1—C2—O3	−171.83 (18)	O3—C3—C8—C7	178.09 (19)
C2—O3—C3—C4	−172.77 (18)	C4—C3—C8—C7	0.2 (3)
C2—O3—C3—C8	9.3 (3)	C6—C7—C8—C3	−0.2 (3)
O3—C3—C4—C5	−178.35 (19)	C5—C6—C9—C10	−114.2 (2)
C8—C3—C4—C5	−0.3 (3)	C7—C6—C9—C10	64.2 (3)
C3—C4—C5—C6	0.4 (3)	C6—C9—C10—O4	−106.1 (2)
C4—C5—C6—C7	−0.3 (3)	C6—C9—C10—O5	72.4 (2)
C4—C5—C6—C9	178.15 (19)		

### Hydrogen-bond geometry (Å, °)

**Table:**

*D*—H···*A*	*D*—H	H···*A*	*D*···*A*	*D*—H···*A*
O2—H2···O4^i^	0.82	1.81	2.617 (2)	168
O5—H5···O1^ii^	0.82	1.85	2.666 (2)	171

Symmetry codes: (i) −*x*+3/2, −*y*+1, *z*+1/2; (ii) *x*+1/2, −*y*+1/2, −*z*+1.
